# Supplementary material for: The genome sequence of the Nut-tree Tussock moth, Colocasia coryli (Linnaeus, 1758) (Lepidoptera: Noctuidae)
Source: Wellcome Open Res. 2025 Dec 23;10:695. [Version 1] doi: 10.12688/wellcomeopenres.25394.1 (PMC12859425; doi:10.12688/wellcomeopenres.25394.1)
Supplement: Peer review history

# Reviewer report: Reviewer response for version 1

**DOI:** 10.21956/wellcomeopenres.27978.r143401

**Published:** 2026-01-30

**Authors:** Yash Gupta

**Affiliations:** 1 Department of Biology, Faculty of Science, Naresuan University, Phitsanulok, Thailand

This is a high-quality genome data note that clearly meets the standards expected for Darwin Tree of Life (DToL) outputs. The rationale is well articulated, methodologies are robust and appropriate, and the resulting datasets are accessible and reusable.

 The manuscript is technically sound and suitable for indexing as a reference genome resource.

Are sufficient details of methods and materials provided to allow replication by others?

Yes

Is the rationale for creating the dataset(s) clearly described?

Yes

Are the datasets clearly presented in a useable and accessible format?

Yes

Are the protocols appropriate and is the work technically sound?

Yes

Reviewer Expertise:

I am a bioinformatician working with insect genomes.

I confirm that I have read this submission and believe that I have an appropriate level of expertise to confirm that it is of an acceptable scientific standard.

## References

[1] : Phylogenetic and mitochondrial genome analysis of a putative new cave cricket species (Rhaphidophoridae) from a Thai subterranean habitat. *Biodiversitas Journal of Biological Diversity* .2025;26(8) : 10.13057/biodiv/d260817 10.13057/biodiv/d260817

# Reviewer report: Reviewer response for version 1

**DOI:** 10.21956/wellcomeopenres.27978.r143397

**Published:** 2026-01-19

**Authors:** David EK Ferrier

**Affiliations:** 1 University of St Andrews, Scotland, UK

The rationale is clear.  This moth has been sequenced as part of the DToL initiative.

 The various protocols are certainly appropriate and the work is of a high technical standard.

 The materials and methods descriptions meet accepted standards for these data notes, and the datasets such as the assemblies of the two haplotypes are accessible and useable.

Are sufficient details of methods and materials provided to allow replication by others?

Yes

Is the rationale for creating the dataset(s) clearly described?

Yes

Are the datasets clearly presented in a useable and accessible format?

Yes

Are the protocols appropriate and is the work technically sound?

Yes

Reviewer Expertise:

NA

I confirm that I have read this submission and believe that I have an appropriate level of expertise to confirm that it is of an acceptable scientific standard.
